# Editorial: Vascular dysfunction and endocrine disorders

**DOI:** 10.3389/fendo.2025.1714212

**Published:** 2025-10-17

**Authors:** Hong Liu, Zhice Xu

**Affiliations:** ^1^ Wuxi Maternity and Child Health Care Hospital & First Affiliated Hospital of Soochow University, Jiangsu, China; ^2^ Department of Anesthesiology and Pain Medicine, University of California Davis Health, Sacramento, CA, United States

**Keywords:** endocrine disruptors, endocrine regulation, endocrine disorders, blood vessels, vascular dysfunction

The intricate interplay between the vascular system and endocrine regulation is essential for maintaining organismal homeostasis. Endothelial and smooth muscle cells—core components of the vascular system—bear receptors for hormones and endocrine factors, enabling bidirectional crosstalk that modulates vascular tone, metabolism, and systemic health. When this crosstalk is disrupted, it triggers a cascade of pathologies affecting both systems, underscoring the need for integrated research into vascular dysfunction and endocrine disorders. This Research Topic, “*Endothelial Dysfunction and Endocrine Diseases*”, collects 17 articles that advance our understanding of the complex interactions between these systems, shedding light on novel mechanisms, risk factors, and potential therapeutic targets across diverse clinical contexts. Below, we summarize key findings from these contributions, highlighting their collective relevance to basic science and clinical practice.

## Metabolic disturbances: linking endocrine imbalances to vascular risk

A central theme in this Research Topic is the role of metabolic dysfunction as a bridge between endocrine disorders and vascular pathology. Two meta-analyses and multiple observational studies dissected this relationship, starting with the association between metabolic syndrome (MetS) and subclinical hypothyroidism (SCH)—a common endocrine disorder linked to ischemic heart disease. A systematic review and meta-analysis by Zhong et al. found that MetS is associated with a 2.56-fold increased risk of SCH (95% CI 1.44–2.55). However, none of the individual components of MetS (e.g., hypertension or dyslipidemia) showed significant associations with SCH. This suggests that the synergistic metabolic perturbations of MetS, rather than isolated factors, drive the risk of SCH —emphasizing the need for holistic metabolic management in at-risk populations.

Complementing this, studies on lipid metabolism markers further connected endocrine and vascular health. One cross-sectional analysis by Tai et al., which used NHANES data from 2005 and 2018, demonstrated that the residual cholesterol-to-high-density lipoprotein cholesterol ratio (RC/HDL-C) is strongly positively associated with hyperuricemia: each 1-unit increase in RC/HDL-C raised the risk of hyperuricemia by 98%. Another NHANES-based study focused on adult men and revealed that the atherogenic index of plasma (AIP)—a marker of lipid metabolism—correlates with testosterone deficiency (TD): each unit increase in AIP was linked to a 2.81-fold higher risk of TD. These findings highlight how lipid-derived biomarkers could serve as predictive tools for endocrine-vascular comorbidities, guiding early intervention.

Vitamin D deficiency emerges as another critical metabolic driver of vascular-endocrine dysfunction. A preclinical study by Kamel et al. showed that vitamin D deficiency exacerbates MetS by impairing endothelial function, increasing oxidative stress (via elevated malondialdehyde), and promoting vascular inflammation. Rats with combined vitamin D deficiency and MetS exhibited thicker aortic walls, increased heart weight, and abnormal vascular reactivity—findings that translate to human health by identifying vitamin D supplementation as a potential strategy to mitigate MetS-related vascular damage.

## Reproductive endocrinology: pregnancy, hormones, and long-term vascular health

Several articles focus on reproductive endocrine contexts, where hormonal fluctuations during pregnancy and menopause exert profound, often long-lasting effects on vascular function. A cross-sectional study by He et al., using NHANES data from 2013 to 2014, found that lower serum estradiol (E2) levels correlate with a higher prevalence of abdominal aortic calcification (AAC) in postmenopausal women. Women in the lowest E2 tertile (2.12–3.57 pg/mL) had a 2.55-fold higher risk of prominent AAC (Kauppila score >5) compared to those in the highest tertile (7.06–38.4 pg/mL). This reinforces the role of E2 in maintaining cardiovascular health in postmenopausal women and supports E2 monitoring for the early prevention of AAC.

Pregnancy-related endocrine disorders, such as gestational diabetes mellitus (GDM) and preeclampsia, were also explored for their vascular impacts. A review by Zhang et al. synthesized evidence showing that GDM disrupts maternal cardiovascular function, umbilical-placental perfusion, and fetal blood flow through mechanisms including endothelial dysfunction, insulin resistance, and epigenetic modifications. These effects increase the long-term cardiovascular risk for both mothers and their offspring, highlighting the need for postpartum vascular surveillance in GDM patients.

Preeclampsia—characterized by pregnancy-induced hypertension—was the focus of a transcriptomic study by Xu et al., which for the first time analyzed gene expression in pure placental microvessels (rather than in whole placental tissue). The study identified 486 differentially expressed transcripts, with hub genes (e.g., ELMO1, YWHAE, and IL6ST) down-regulated in preeclamptic small blood vessels. Functional tests revealed blunted vasoconstriction to angiotensin II and reduced vasodilation to nitric oxide donors in preeclamptic blood vessels—findings that suggest novel molecular targets for preeclampsia’s vascular pathology.

Prenatal exposure to glucocorticoids (GCs) is another key reproductive factor linked to offspring vascular dysfunction. A review by Q. Gao et al. explained that while placental 11β-hydroxysteroid dehydrogenase 2 normally protects fetuses from maternal GCs by inactivating cortisol, adverse prenatal factors (e.g., stress, caffeine, synthetic GC use) can overwhelm this barrier. Excessive fetal GC exposure leads to long-term cardiovascular issues in offspring, including hypertension and impaired vascular function—underscoring the need for cautious GC use in pregnancy.

## Diabetes and its vascular complications: from cognitive impairment to macrovascular disease

Type 2 diabetes mellitus (T2DM)—a hallmark endocrine-metabolic disorder—was the focus of three articles, each exploring distinct vascular sequelae. A pilot study by Hou et al. used 3D-arterial spin labeling (3D-ASL) to measure cerebral blood flow (CBF) in T2DM patients with mild cognitive impairment (MCI). Compared to T2DM patients without MCI, those with MCI had significantly lower CBF in the temporal, parietal, occipital, and hippocampal regions. Hippocampal CBF exhibited the greatest diagnostic efficacy for MCI (AUC = 0.813), positioning 3D-ASL as a promising tool for early MCI detection in patients with T2DM.

Another study (by Wang et al.) investigated Isthmin-1 (ISM-1)—a novel adipokine—in T2DM patients with macrovascular complications (MACV). Serum ISM-1 levels were highest in MACV patients, followed by T2DM patients without MACV, and lowest in healthy controls. ISM-1 positively correlated with blood pressure, triglycerides, HbA1c, and insulin resistance (HOMA-IR), suggesting that it may contribute to macrovascular disease by disrupting glucose-lipid metabolism.

Acute ischemic stroke (AIS) in T2DM patients was explored in a retrospective study by (Wang et al.). The authors found that HbA1c >6.5% is associated with severe hypercoagulability and heightened inflammation (via markers like the systemic immune-inflammation index, SII). An HbA1c level >6.5% was an independent predictor of hypercoagulability (OR = 1.74), linking chronic hyperglycemia to thromboinflammation in AIS—findings that support the idea that tight glycemic control improves stroke outcomes.

## Therapeutic insights and emerging targets

This Research Topic also offered critical insights into therapeutic interventions for vascular-endocrine disorders. For example, a network meta-analysis (by Keng et al.) of 18 randomized controlled trials (RCTs) compared six interventions for thin endometrium—a common cause of infertility in assisted reproductive technology (ART). Platelet-rich plasma (PRP) ranked highest for improving clinical pregnancy rates (SUCRA = 80.12%) and was among the top three for increasing endometrial thickness (SUCRA = 68.14%), making it a promising first-line option for ART patients.

In atherosclerosis—driven by endothelial dysfunction—a review by Yan et al. categorized adipokines into protective (e.g., adiponectin, FGF21, and CTRP9) and detrimental (e.g., leptin, resistin, and FABP4) subsets. Targeting adipokines by enhancing protective adipokine signaling or inhibiting detrimental ones could offer novel therapies for atherosclerosis-related cardiovascular disease (CVD).

Finally, a review by Lan et al. expanded the scope of vasoactive agents—traditionally used for cardiovascular regulation—by exploring their non-cardiovascular effects. Agents such as angiotensin II and vasopressin influence endocrine functions (e.g., insulin secretion), the central nervous system, and gastrointestinal motility, emphasizing the need to monitor off-target effects in clinical practice.

## Future directions

This Research Topic underscores the interconnectedness of vascular dysfunction and endocrine disorders, spanning metabolic syndrome, reproductive health, diabetes, and beyond. Key takeaways include the identification of novel biomarkers (e.g., RC/HDL-C, AIP, and hippocampal CBF), the role of hormonal fluctuations (e.g., E2 and GCs) in vascular health, and promising therapeutics (e.g., PRP, vitamin D supplementation, and adipokine targeting).

Future research should prioritize longitudinal studies to confirm causal relationships (e.g., between RC/HDL-C and hyperuricemia) and large-scale RCTs to validate interventions such as PRP for thin endometrium. Additionally, integrating multi-omics approaches—such as transcriptomics (e.g., preeclamptic placental microvessels) and microbiomics (emerging in endometriosis research)—will deepen our understanding of the underlying mechanisms.

By bridging the gap between basic science and clinical practice, this Research Topic provides a foundation for personalized strategies to prevent, diagnose, and treat vascular-endocrine comorbidities—ultimately improving patient outcomes and advancing the field of integrative cardiovascular-endocrine medicine.

